# Analyzing the Interaction between Two Different Types of Nanoparticles and Serum Albumin

**DOI:** 10.3390/ma12193183

**Published:** 2019-09-28

**Authors:** Roxana E. Cristian, Israa J. Mohammad, Maria Mernea, Beatrice G. Sbarcea, Bogdan Trica, Miruna S. Stan, Anca Dinischiotu

**Affiliations:** 1 Department of Biochemistry and Molecular Biology, Faculty of Biology, University of Bucharest, 91-95 Spl. Independentei, 050095 Bucharest, Romania; roxana.cristian95@yahoo.com (R.E.C.); israa.biochem88@gmail.com (I.J.M.); anca.dinischiotu@bio.unibuc.ro (A.D.); 2 Department of Anatomy, Animal Physiology and Biophysics, Faculty of Biology, University of Bucharest, 91-95 Spl. Independentei, 050095 Bucharest, Romania; maria.mernea@bio.unibuc.ro; 3 Materials Characterization Department, National Institute for Research & Development in Electrical Engineering (ICPE-CA), 313 Splaiul Unirii, 030138 Bucharest, Romania; gabi_bea@yahoo.com; 4 National Institute for Research & Development in Chemistry and Petrochemistry (INCDCP-ICECHIM), 202 Spl. Independentei, 060021 Bucharest, Romania; trica.bogdan@gmail.com; 5 Department of Science and Engineering of Oxide Materials and Nanomaterials, Faculty of Applied Chemistry and Materials Science, University Politehnica of Bucharest, 1-7 Gheorghe Polizu Str., 011061 Bucharest, Romania

**Keywords:** biocorona, nanoparticles, serum albumin, silica, magnetite, titanium dioxide

## Abstract

Two different types of nanoparticles (silicon dioxide and titanium dioxide) were selected within this study in order to analyze the interaction with bovine and human serum albumin. These particles were characterized by transmission and scanning electron microscopy (TEM and SEM), X-ray diffraction (XRD) and energy dispersive X-ray spectroscopy (EDXS). In addition, the hydrodynamic size and the zeta potential were measured for all these nanoparticles. The serum proteins were incubated with the nanoparticles for up to one hour, and the albumin adsorption on the particle surface was investigated by sodium dodecyl sulfate-polyacrylamide gel electrophoresis (SDS-PAGE). The effect induced on the secondary structure of proteins was analyzed by Fourier transform infrared spectroscopy (FTIR). The results showed that albumin adsorbed on the surface of both types of nanoparticles, but in different quantities. In addition, we noticed different changes in the structure of albumin depending on the physicochemical properties of each type of particle tested. In conclusion, our study provides a comparative analysis between the different characteristics of nanoparticles and the protein corona formed on the particle surface and effects induced on protein structure in order to direct the development of “safe-by-design” nanoparticles, as their demands for research and applications continue to increase.

## 1. Introduction

The huge enthusiasm for nanotechnology and the wide use of nanomaterials (NMs) have a strong impact on the development of world nanomaterials market, which was evaluated by Mordor Intelligence at about USD 4.1 billion in 2015 [1] and it is optimistically expected by Accuray Research LLP to be worth over US$173 billion globally by 2025 [2]. The constant growth of engineered nanomaterials (ENM) production will certainly influence the ENMs release into the environment during manufacture, transport, use, and disposal, which will trigger unknown risks to human health and the environment. Thus, the toxicity of NMs, presence of toxic solvents during the synthesis, release of hazardous intermediate compounds, and toxicity of wastes that resulted from NMs’ processing or manufacturing can represent the major factors that will affect the development of the global nanomaterials market [3]. 

According to the Allied Market Research experts’ team, the metal and non-metal oxide-based NMs are the most widely produced and consumed NMs, on top being titanium dioxide (TiO_2_), followed by silicon dioxide (SiO_2_), and then by other oxide-based NMs, such as iron oxides and zinc oxide (ZnO) [4]. 

SiO_2_ nanoparticles (NPs) are frequently used as anti-caking, anti-foaming or fluidizing agents in powdered foods. Moreover, silica can be utilized as a clearing agent in juices, oil and the beer industry or as a flavoring agent [5]. The amount of silica ingested daily was estimated based on a Dutch food consumption survey to be 9.4 mg per kg body weight of an average adult, of which 1.8 mg/kg body weight/day fell within the nanoparticle range [6]. Furthermore, an increase of silica NPs in cosmetic products is anticipated, as these can be used in products for hair, skin, lips, face and nails. 

The surface of silica NPs can be easily modified with functional molecules, including antibodies for efficient targeting and fluorophores for imaging [7], and can be loaded with a variety of molecules, such as drugs, peptides and therapeutic proteins, but also genes, in order to be used as drug delivery systems [8]. One of the bio-medical applications of SiO_2_ NPs is the separation of proteins and their adsorption in a biological medium. Studies have shown that SiO_2_ NPs conjugated to polypyrrole ester derivatives can interact with human serum albumin (HSA) forming covalent bonds, the interaction between the two being evidenced by incubation with anti-HSA antibodies [9].

TiO_2_ NPs are frequently used as photocatalysts as these possess relatively high photocatalytic activity, surface-to-volume ratio and chemical stability. These NPs are extensively used for the removal of micropollutants in water treatment [10], for self-cleaning and antibacterial efficiency in textile fibers, and for the ability to block UV radiation while remaining transparent on the skin in sunscreens. Although more than 10,000 tons of TiO_2_ are produced and imported into France each year, the French Ministry of Ecological and Solidarity Transition has decided to ban the placing on the market of foodstuffs containing titanium dioxide as from 1 January 2020 based on toxicity studies conducted between 2017 and 2019, which were not able to ensure the safety of TiO_2_, recommending the limitation of consumer exposure to this substance, in particular by substituting these nanomaterials with safe and equivalent products [11].

Almost all uptake routes associated with NP exposure (skin pores, debilitated tissues, injection, olfactory, respiratory and intestinal tracts) [12] will be followed by the contact with blood vessels inside the human body. Transport of NPs through the blood is favored by the hydrodynamic forces of blood flow [13], and their interaction with blood proteins that occurs immediately after injection [14]. Once within the biological media, NPs will be targets for proteins or other biological macromolecules that will compete to bind to their surface. Thus, NP-protein complexes will influence the cellular behavior, biological activity and biodistribution of nanoparticles, being very important elements in testing the NPs toxicity [15].

The composition and molecular properties of the protein corona have been shown to be influential factors for cellular uptake of NPs, but the direct relation between structure and effect remains to be established [16]. Although biocorona is an extremely dynamic structure and its composition changes over time, human serum albumin (HSA), immunoglobulins and fibrinogen have been shown to be the main proteins in the "hard" corona component, which bind tightly to the nanoparticle surfaces and exhibit distinctive stability. 

In order to provide a comparative analysis between the different characteristics of NPs and the effects induced on the structure of protein adsorbed onto the particle surface, we selected for our study two different types of NPs (SiO_2_ and TiO_2_) which were characterized by transmission and scanning electron microscopy (TEM and SEM), scanning electron X-ray diffraction (XRD), energy dispersive X-ray spectroscopy (EDXS), hydrodynamic size and the zeta potential measurements. In addition, the interaction with bovine serum albumin (BSA) and HSA was investigated by sodium dodecyl sulfate-polyacrylamide gel electrophoresis (SDS-PAGE) and by Fourier transform infrared spectroscopy (FTIR). 

## 2. Materials and Methods 

### 2.1. Nanoparticles 

SiO_2_ NPs were obtained from NaBond Technologies Co., Ltd. (Shenzhen, China), being synthesized by the reactive laser ablation method. TiO_2_ Degussa P25 powder was purchased from Sigma-Aldrich (St. Louis, MO, USA), having a surface area of 35–65 m^2^/g (Brunauer-Emmett-Teller, BET). All nanoparticles were homogenized in Milli-Q water at a concentration of 1 mg/ml.

### 2.2. Nanoparticle Characterization 

The NP characteristics were analyzed using transmission and scanning electron microscopy (TEM and SEM), and the hydrodynamic size and zeta potential were quantified. 

The NPs were dispersed in distilled water and small drops were added consecutively on top of a pure carbon film copper grid (Ted Pella, Inc., Redding, CA, USA). Each time, the excess was carefully removed using filter paper. The sample was analyzed in bright field mode using a Tecnai F20 G2 TWIN Cryo-TEM (FEI, Hillsboro, OR, USA) at an acceleration voltage of 200 kV, and subjected to energy-dispersive X-ray spectroscopy (EDXS) (Oxford Instruments, Abingdon, UK). 

SEM investigations were performed on Zeiss Auriga microscope (Carl Zeiss, Jena, Germany). Images were acquired at a voltage of 2 kV and contrast-enhanced for better observation. The samples were analyzed using the D8 Discover X-ray diffractometer (Bruker AXS, Karlsruhe, Germany), Cu-anode X-ray tube (40 kV, 40 mA) (Bruker AXS, Karlsruhe, Germany) and LYNX EYE detector (Bruker AXS, Karlsruhe, Germany). The crystallite size was calculated using the Debye–Scherer formula. 

The hydrodynamic size and zeta potential of NPs (100 µg/mL) suspended in ultrapure Milli-Q water was assessed by dynamic light scattering (DLS) and electrophoretic light scattering (ELS), respectively, using a Malvern Nano-ZS instrument (Malvern Instruments, Malvern, Worcestershire, UK). The measurements were performed in triplicate at 25 °C. 

### 2.3. SDS-PAGE

The level of protein adsorbed on the surface of NPs was evaluated by SDS-PAGE. A concentration of 100 μg/ml NPs was incubated with 1 mg/ml BSA or HSA prepared in PBS at 37 °C for 10 min, 30 min and 60 min. At the end of each time interval of incubation, the suspensions were centrifuged at 14,000 × g for 10 min. A volume of 6 µl of the supernatants consisting of proteins which were not adsorbed onto NPs were mixed with sample loading buffer. All the content was loaded with a Hamilton syringe on a 10% SDS-PAGE and run under reducing conditions. The gels were stained using in 0.025% w/w Coomassie Brilliant Blue dispersed in 10% ethanol and 10% acetic acid. The gel was left overnight to stain before imaging. The bands were visualized on a ChemiDoc MP Imaging system (Bio-Rad, Hercules, CA, USA) and were quantified using the Image Lab 5.0 software (Bio-Rad, Hercules, CA, USA). BSA and HSA (0.25–4 μg protein) calibration curves were obtained by SDS-PAGE and these were used in order to calculate the amount of protein adsorbed on NPs. This amount represents also the quantity of protein loaded on gel which was measured before the SDS-PAGE by Lowry protein assay as a standard spectrophotometric method. All the samples were run at least in triplicate.

### 2.4. FTIR Measurements

A Bruker Tensor 27 spectrometer (Bruker Optik GmbH, Ettlingen, Germany) was used to perform FTIR attenuated total reflection (ATR) measurements on HSA, BSA, HSA-NPs and BSA-NPs samples. HSA and BSA were incubated with NPs in 1:1 ratio for 15, 30, and 60 min. Protein-NPs suspensions were centrifuged and the sediment was dried. The resulting powder was deposited on the ATR crystal in order to perform the measurement by considering the following parameters: (i) addressed frequency range: 4000–400 cm^−1^; (ii) resolution: 4 cm^−1^ and (iii) collection time: 1 min. Spectra were further analyzed (smoothing, normalization, peaks identification and characterization) using OPUS software (Version 7.2, Bruker Optik GmbH, Ettlingen, Germany).

## 3. Results

### 3.1. Morphological and Physico-Chemical Characterization of NPs

The NPs characteristics were analyzed using transmission and scanning electron microscopy (TEM), energy-dispersive X-ray spectroscopy (EDXS), and zeta potential analyses. 

**Figure 1:**
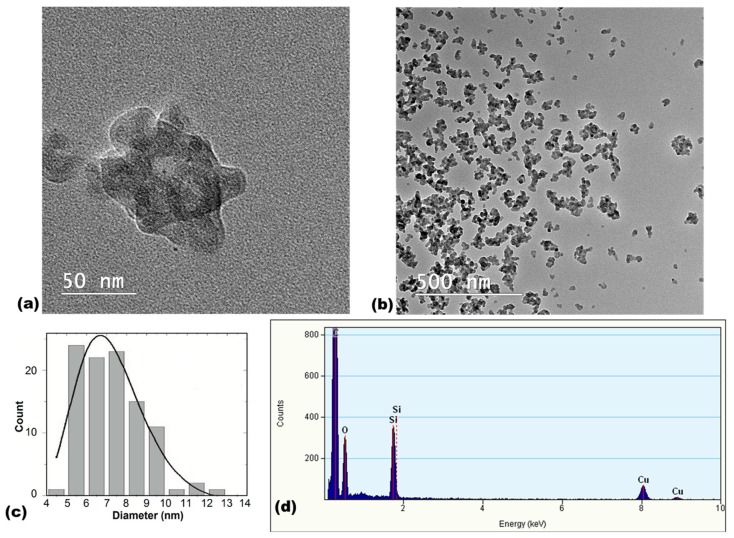
Characterization of SiO_2_ NPs by TEM (scale bar of 500 nm (**a**) and scale bar of 50 nm (**b**)), size distribution (**c**) and energy-dispersive X-ray spectroscopy (EDXS) (**d**) analyses.

TEM analysis showed that SiO_2_ NPs have a spherical aspect and are very similar in size, measuring less than 10 nm in diameter (Figure 1a). The size distribution obtained based on a statistic from TEM images confirmed this estimation as it was a lognormal function with values between 4 and 13 nm and an average size of about 7 nm (Figure 1c). The silica NPs have the tendency to form agglomerates between 50–300 nm with variable shapes according to Figure 1a,b). 

**Figure 2:**
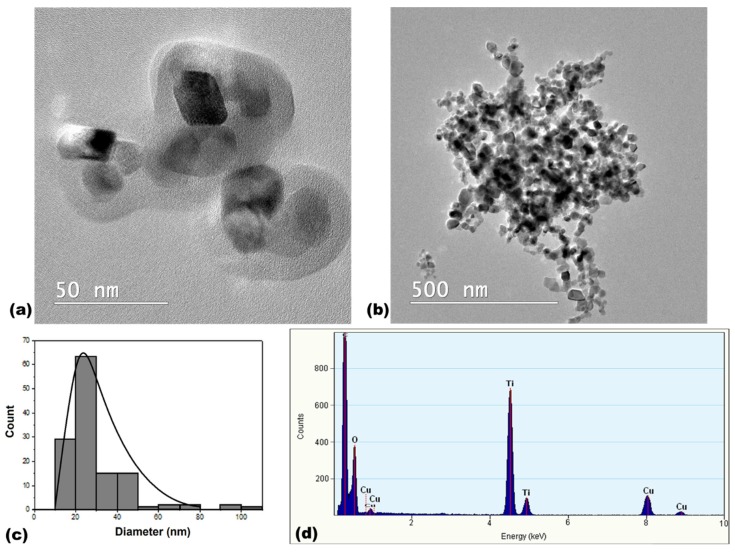
Characterization of TiO_2_ NPs by TEM (scale bar of 500 nm (**a**) and scale bar of 50 nm (**b**)), size distribution (**c**) and energy-dispersive X-ray spectroscopy (EDXS) (**d**) analyses.

We can observe the ellipsoidal morphology (bar-like shape) of TiO_2_ NPs (Figure 2a) and their distribution in agglomerates suggesting that these particles are not uniformly distributed at nanometric level (Figure 2b). The agglomerates of TiO_2_ NPs were much bigger that those observed for silica NPs, an estimation from TEM images (Figure 2b) confirming a cluster’s size higher than 500 nm. The particles formed these large agglomerates due their small size and high relative surface, which were specific features for titanium dioxide. The P25 particles have a polyhedral shape with round corners. A mean size of 29 ± 15 nm was calculated based on the graph of particle size distribution shown in Figure 2c.

Scanning transmission electron microscopy (STEM) was required to determine elemental composition by energy-dispersive X-ray spectroscopy (EDXS) determination, which revealed the presence of silicon and oxygen in a proportion of 1:2 (Figure 1d), confirming the pure SiO_2_ composition for silica NPs, and the presence of titanium and oxygen in a 1:2 ratio for TiO_2_ NPs (Figure 2d). 

**Figure 3:**
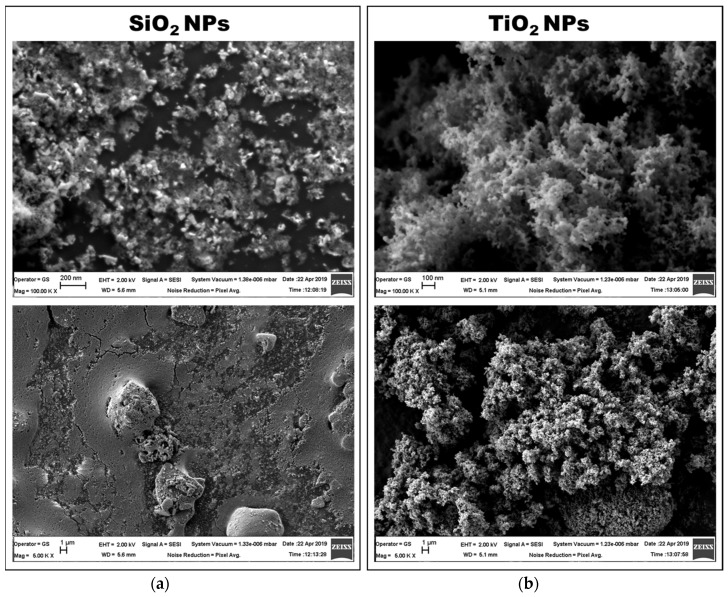
Characterization of SiO_2_ (**a**) and TiO_2_ (**b**) NPs by SEM (top—100,000× magnification and bottom—5000× magnification).

The analysis of SEM images taken with 100,000× magnification revealed the clusters of NPs that were organized in different shapes for each type of particle studied (Figure 3). For SiO_2_ NPs, the clusters were formed in the range of nano size, less than 200 nm, based on the scale bar of the images. A granular distribution of SiO_2_ particles can be observed in the images with 100,000× magnification (Figure 3a-top). The TiO_2_ NPs agglomerated in a coral-like shape, allowing the formation of a well-organized 3D structure. Furthermore, an overview of particles’ arrangement was observed by SEM with 5000× magnification (Figure 3b-bottom). Compared to TiO_2_ NPs, the silica NPs distributed in a much more uniform layer, with few large spheres of NPs agglomerated. 

**Figure 4:**
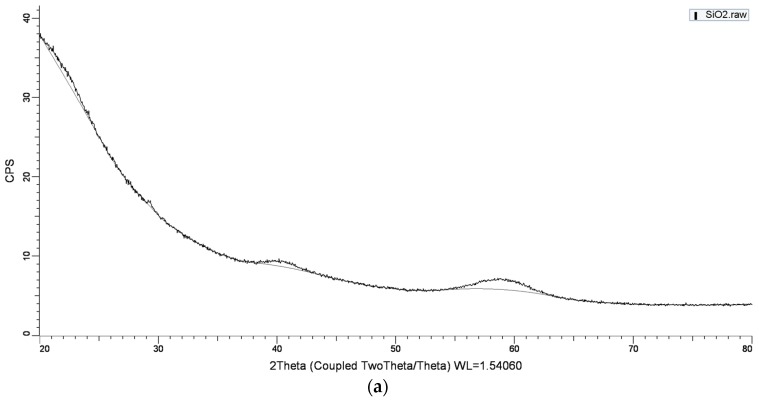

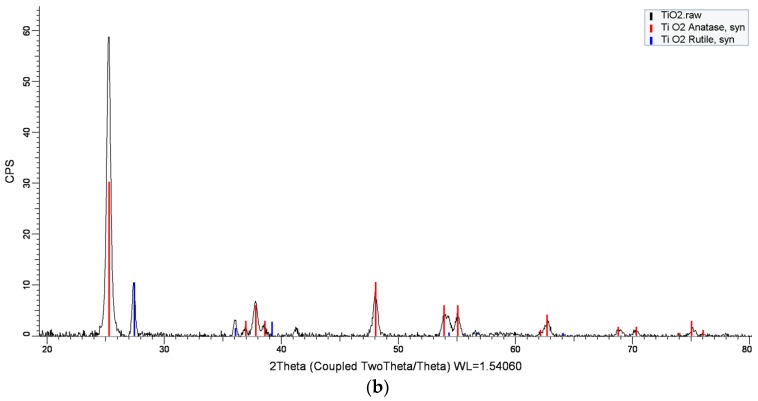
Characterization of SiO_2_ (**a**) and TiO_2_ (**b**) NPs by XRD.

The XRD pattern of SiO_2_ NPs (Figure 4a) indicated that the amorphous structure of the material is based on the highest and broad peak at 2θ value of 20° [17]. In addition, the broad XRD peaks indicate the formation of nanosized particles.

In the case of TiO_2_ NPs, the XRD profile (Figure 4b) showed the characteristics peaks of two nanoscaled phases: anatase at 2θ values of 25.21°, 37°, 37.9°, 48.1°, 54°, 55°, 62.7°, 68.9°, 70.1° and 75.1°, and of rutile at 2θ values of 27.6°, 36.2°, 39.2° and 54.3°. These results confirmed an anatase/rutile weight ratio of about 83/17, as it was previously reported [18].

DLS represents standard practice over the past decade for measuring the size and dispersity of NPs. Silica NPs had the lowest diameter (134.4 nm) compared to TiO_2_ NPs, suggesting a better dispersion in water for them as confirmed by the polydispersion index of 0.255 (Table 1). The obtained value was in agreement with the size of clusters revealed on SEM images. Compared to silica, the average hydrodynamic size of TiO_2_ NPs was higher than 1300 nm due to the presence of large and sedimentary particles. Due to this issue, we decided not to use this analysis in order to check the protein adsorption on particles or to perform the measurements in another medium than distilled water, as the results could not be reliable for providing correct and valid information. Furthermore, zeta potential was measured to estimate the effective electric charge on the NP surface (Table 1). The average value of zeta potential determined for SiO_2_ NPs was almost similar (−24.4 mV), which confirmed that the electrostatic stabilization of these particles dispersed in water. For TiO_2_ NPs, the value of zeta potential was almost half of the other particles, highlighting the difference between them regarding the colloidal stability. 

### 3.2. Protein Adsorbtion on NP Surface

In the search for the best way to quantify the protein binding to nanoparticles, there can be different experimental approaches mentioned that present both advantages and drawbacks. SDS-PAGE is one major technique for the examination of biocorona that can be used alone or in combination with other complementary methods, such as mass spectrometry, FTIR and thermogravimetric studies [19,20]. The common protocol involves several steps of centrifugation or ultracentrifugation in order to sediment the particles and to remove the unbound proteins found in supernatant. The particles are re-dispersed in ultrapure water, SDS or Laemmli sample buffer supplemented with 2-mercaptoethanol and boiled for 5 min at 95 °C in order to elute and denature the proteins adsorbed on the NP surface [21]. This kind of procedure can raise some concerns, including the high probability of not detaching all of the proteins adsorbed during the incubation which trigger unreliable results on SDS-PAGE or liquid chromatography–mass spectrometry (LC-MS). As an alternative, the supernatant with unbound proteins can be used for an indirect evaluation of protein corona. Therefore, after separating the NPs from the solution with protein by centrifugation, the amount of protein bound can be estimated after the reduction of the protein concentration in the supernatant using any convenient protein assay, such as UV absorbance, Bradford or Lowry assay, fluorescence-based method, and SDS-PAGE. 

**Figure 5:**
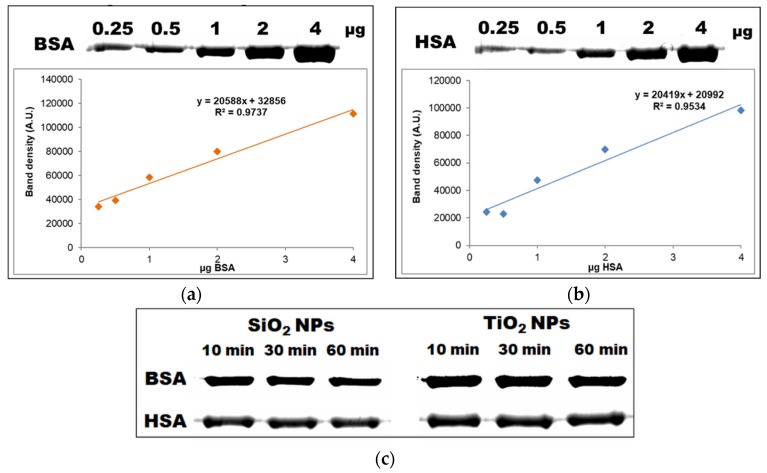

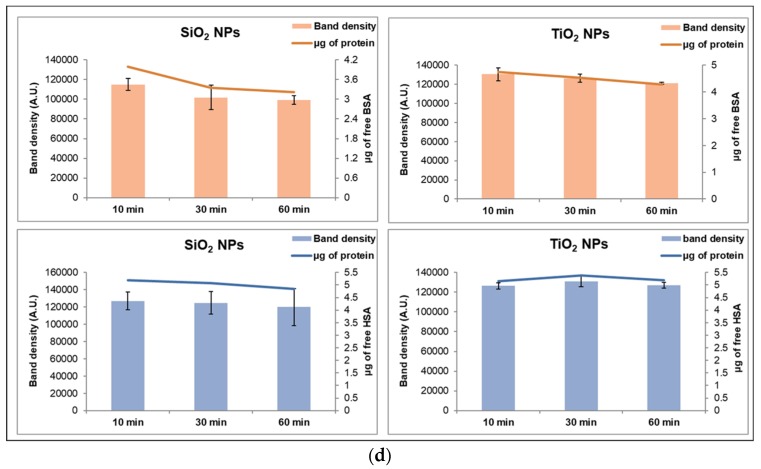
Time evolution of the interaction between serum albumin and nanoparticles (NPs). Representation of bovine serum albumin—BSA (**a**) and human bovine albumin—HSA; (**b**) calibration curves obtained in the range of 0.25–4 μg protein by SDS-PAGE; (**c**) one-dimensional SDS-PAGE of unbound serum proteins (BSA and HSA) obtained after 10, 30 and 60 min of incubation with NPs; (**d**) the amount of unbound BSA and HSA was calculated as μg of protein after extrapolating the band density on the BSA and HSA standard curves. The values are represented as means ± standard deviation (*n* = 3).

Thus, we decided to quantify the amount of unattached protein and to correlate the data with FTIR results where we used the whole pellet of NP-protein. Based on the BSA and HSA standard curves (Figure 5a,b, respectively), the band densities for each incubation time were extrapolated and the amount of free protein was calculated (Figure 5c,d). More significant changes over time were noticed for the incubation with BSA in comparison with HSA samples. A time-dependent decrease of free BSA was noticed in the case of SiO_2_ and TiO_2_ NPs, indicating actually an increase in BSA adsorption on their surface during the incubation. In the case of unbound HSA, a slight decrease in time was observed for SiO_2_ NPs, and almost no changes for TiO_2_ NPs. Taken together, we can notice almost the same trend for both proteins for each type of NP. Furthermore, the amount of unbound protein in the supernatants was higher for HSA samples, suggesting that the adsorption on NPs surface was much more intense for BSA, and the affinity and dynamics of HSA for the NPs surface were lower compared to bovine protein. 

### 3.3. Influence Triggered by NPs on the Secondary Structure of Proteins

**Figure 6:**
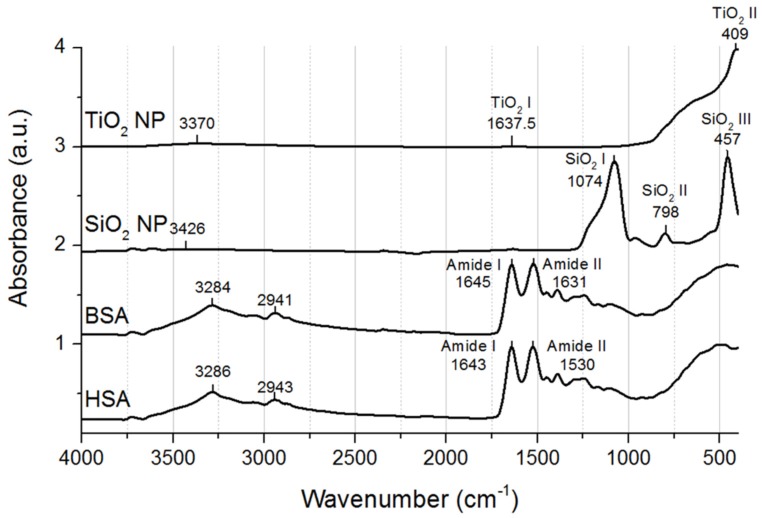
FTIR spectra of HSA, BSA, SiO_2_ NPs and TiO_2_ NPs. Spectra are presented with Y offsets. The main absorption bands and their central frequencies are labelled on the plots.

FTIR was previously used to investigate protein–NP interactions, as the absorption bands visible on the spectra are molecule-specific and give information on the composition of samples [22,23,24]. Proteins present several absorption bands in IR spectral range, from which amide bands (A, B, I, II, III) result from the vibration of the backbone. Amide A (~3300 cm^−1^) and B (~3070 cm^−1^) arise from NH stretching vibrations and are not influenced by the conformation of the protein backbone. Amide I band (~1650 cm^−1^) resulting mostly from C=O stretching vibration and amide II band (~1550 cm^−1^) resulting mostly from an out-of-phase combination of NH in plane bending and CN stretching vibrations are strongly influenced by backbone structure. Amide III band (1400–1200 cm^−1^), reflecting mostly the in-phase combination of NH bending, NH in plane bending and CN stretching vibrations are complex as they are influenced by backbone and side chain structure [24]. In Figure 6, we present the FTIR spectra of HSA and BSA. Amide I and amide II bands, as well as their central frequencies, are labelled in the figure. Given the sole influence of backbone structure on these bands, we decided to use them for further analysis of HSA or BSA binding to NPs.

The spectra of NPs are also plotted in Figure 6. The spectrum of SiO_2_ NPs presents three distinctive absorption bands around 3426 cm^−1^, 1074 cm^−1^ (SiO_2_ I), 798 cm^−1^ (SiO_2_ II) and 457 cm^−1^ (SiO_2_ III), assigned to O-H stretching vibration (3426 cm^−1^), Si-O-Si asymmetric and symmetric stretching modes (SiO_2_ I and II) and to the bending mode (SiO_2_ III) [25]. The spectrum of TiO_2_ NPs present absorption bands around 3370 cm^−1^, 1637.5 cm^−1^ (TiO_2_ I) and 409 cm^−1^ (TiO_2_ II), which can be assigned to hydroxyl group stretching vibrations (first band) or to Ti-OH bending modes (TiO_2_ I) [26]. As can be seen in Figure 1, the band TiO_2_ I overlaps amide I bands of proteins. 

**Figure 7:**
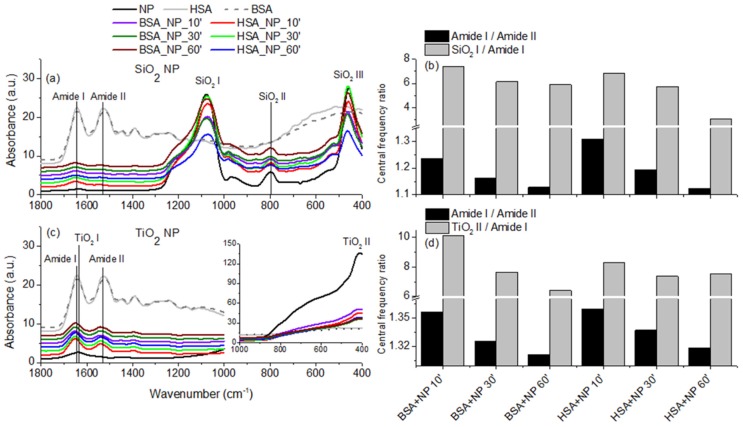
(**a**,**c**) FTIR spectra of HSA, BSA, nanoparticles (NP), HSA incubated with NPs for 10 min (HSA_NP_10’), 30 min (HSA_NP_30’) and 60 min (HSA_NP_60’) and BSA incubated with NPs for 10 min (BSA_NP_10’), 30 min (BSA_NP_30’) and 60 min (BSA_NP_60’). Results for SiO_2_ NPs are presented in (**a**), for TiO_2_ NPs are presented in (**c**). The main absorption bands are labelled on every plot. (**b**,**d**) ratio between the central frequencies of some absorption bands from the spectra of proteins incubated with SiO_2_ NPs (**b**) and TiO_2_ NPs (**d**).

We focused on the analysis of the spectra of proteins incubated with NPs to the 1800–400 cm^−1^ frequency range as this region comprises the main absorption bands of both proteins and NPs. The spectra are presented in Figure 7a,c,e. As it can be seen, all spectra of proteins and proteins incubated with NPs present the amide I and II bands. Additionally, the bands specific to NPs can be identified in the spectra of proteins incubated with NPs, evidence that proteins were bound to NPs. For the analysis, we considered the shift in the central frequencies of absorption bands in in the ratio between the maximum absorbance of some bands. 

In Table 2, we present the central frequencies of amide I and amide II bands of proteins and the representative bands of NPs. In comparison to the pure proteins, we observe that the central frequencies of proteins incubated with NPs shift to higher wavenumbers in the case of samples comprising SiO_2_ and TiO_2_ and to lower wavenumbers in the case of proteins incubated with TiO_2_. Since both amide I and amide II bands are sensitive to the conformation of the proteins and to the secondary structure composition of the proteins, the shift of amide bands suggest that proteins underwent a conformational change upon the binding to NPs. The secondary structures of proteins present absorptions within the amide I band frequency range, the central frequencies of the bands associated with α-helices being ~1645 cm^−1^ or with β-sheets being ~1633 and 1684 cm^−1^ [24]. The shifts observed in the amide I bands of proteins incubated with NPs point toward an increase in proteins helicity due to the interaction with SiO_2_ and TiO_2_ NPs. SiO_2_ NPs do not present an absorption band overlapping the amide band of proteins; therefore, the shift can be explained only by the conformation transition of proteins. In addition, the SiO_2_ I absorption band shifted to lower wavenumbers, evidence of the interaction of NPs with proteins. In the case of sample comprising TiO_2_ NPs, the amide I band of proteins overlaps TiO_2_ I band of NPs. Even if TiO_2_ I central frequency is at a lower wavenumber (1637.5 cm^−1^), the amide I band of proteins incubated with NPs shifts to higher wavenumbers. The TiO_2_ II band shifts from 409 cm to 405 cm^−1^ in the case of the sample incubated with HSA for 10 min or to 411 cm^−1^ in the case of the sample incubated with BSA for 10 min, or remains the same in the case of other samples. 

The differences in the amide I/II bands ratios calculated for the same protein in different conditions are probably due to changes in their secondary and possibly tertiary structures [27]. In order to better characterize the conformation changes of proteins due to the binding to NPs, we also calculated the ratio of protein amide I to amide II bands. Results are presented in Figure 7b,d,f. In the case of pure proteins, the ratio is ~1 for both HSA and BSA. In the case of proteins interacting with SiO_2_ NPs (Figure 7b), the amide I /amide II ratio decreases with incubation time: from 1.23 to 1.13 in the case of BSA and from 1.3 to 1.12 in the case of HSA. In the case of proteins—TiO_2_ NPs interactions (Figure 7d), the ratio amide I/amide II of BSA decreases from 1.35, corresponding to samples incubated for 10 min, to 1.31 corresponding to samples incubated for 60 min. In the case of HSA, the ratio decreases with incubation time, from 1.36 to 1.32. These results show that, due to the interaction with SiO_2_ or TiO_2_ NPs, the conformation of both albumins continuously changes during the incubation time. The same tendency of amide I/amide II ratios, correlated with similar shifts in amide I and amide II bands’ central frequencies, suggest that similar conformational transitions occur when the proteins bind to these NPs. 

The interaction of NPs with BSA and HSA was further characterized by calculating the ratio between the maximum absorbance associated to a band specific to NPs and the maximum absorbance of protein amide I band. We expect this ratio to decrease upon the binding of proteins to NPs, as the intensity of amide I band should increase with the quantity of protein present in the sample, while the intensity of the NP specific band should remain unchanged. Results are presented in Figure 7b,d,f. When considering SiO_2_ NPs, results in Figure 7b show that the ratio presents a continuous decrease in the case of both BSA and HSA. The ratios between TiO_2_ II and amide I bands of BSA present a decrease with incubation time. The TiO_2_ II /amide I band of HSA decreases in the 30 min of incubation, remaining at a similar value after 60 min of incubation. Results obtained in the case of BSA and HSA binding to SiO_2_ and TiO_2_ NPs suggest that the proteins continuously bind to the NPs in increasing amounts with the incubation time. The tendencies of increased adsorption of proteins on the surface of SiO_2_ and TiO_2_ NPs are in agreement with protein dosing results obtained by SDS- PAGE.

## 4. Discussion

Being one of the fastest growing and most promising technologies nowadays, nanotechnology deserves an important amount of attention and evaluation. Although it is not possible to exactly quantify the amounts of nanomaterials being produced and used, the European Union alone invested between 2007 and 2011 approximately EUR 896 million in nanotechnology related research. Therefore, it is understandable that nanotechnology will continue its growth and new topics of study are necessary to fully understand both possibilities and risks (nanomedicine and nanotoxicology, respectively) [28], as nanoparticles could represent a great potential hazard during the manufacturing, during the normal life of the application and during the recycling of the nanomaterial.

Regarding the nanomaterial toxicity data and the characterization of their properties, there is a lack of consistency and it is quite difficult to compare different studies or to reproduce results, as different approaches are undertaken for the toxicity testing of nanomaterials and the full methodology is often not reported. Thus, there is an urgent need for developing laboratory tests that produce results that are representative of nanomaterial toxicity [29].

It is generally accepted that NP biocompatibility is influenced by the size, shape and surface properties of NPs, such as electrical charge, functional groups and the presence of attached molecules [30]. Along with stability, other properties such as NP size, toxicity, total electrical charge, nature of functionalization layer or protein absorption capacity are key elements that are different depending on the method of NP synthesis.

The absorption of proteins by the dispersed nanoparticles in the physiological fluids can occur, and the final opsonized nanoparticles are often captured by macrophages from the reticuloendothelial system [31]. For example, the iron oxide NPs injected intravenously as drug delivery systems are guided to the tissue of interest, subsequently immobilized in the tissues (while the drug is released), and finally eliminated from the body. The biodistribution and pharmacokinetics of intravenously injected nanoparticles are influenced by the size of the nanoparticles. Blood proteins are absorbed in smaller amounts on small particles than large ones, and smaller particles are more difficult to remove from the system than larger ones [32].

The formation of the protein corona can significantly affect the cell uptake of the nanoparticles as it was shown at the level of human plasma for silicon nanoparticles of different sizes and functionalities on the surface [33]. In addition, the presence of BSA can enhance the cellular binding of BSA-cationic NP complexes [34]. Furthermore, agglomeration and surface chemistry seem to be one key to enhanced uptake into cells [35]. 

The two types of particles selected in our study are metal and non-metal oxide NPs with different structure, morphology and size. In order to obtain a useful correlation between their physico-chemical properties and the interaction with pure proteins (because, using human sera, it can exist a lot of inter-individual variability within the biocorona [36], we designed a research study able to minimize the unreliable results or subjected to irreproducibility. First of all, the TEM and SEM images revealed the agglomeration tendency of all NPs, which was confirmed by DLS measurements in ultrapure water. 

Furthermore, we wanted to correlate the surface characterization of NPs conducted using SEM, in terms of shape, homogeneity, surface curvature, topology and volume, with the protein affinity and adsorption onto the surface of particles. Being the most aggregated particles from the beginning, as shown by hydrodynamic size (Table 1) and TEM images (Figure 2) for TiO_2_ NPs, this could explain the lack of dynamics over time in protein adsorption on their surface in comparison with the other type of particles (Figure 5d). The good dispersion of SiO_2_ NPs revealed in TEM images (Figure 1) and by hydrodynamic size and colloidal stability may represent a key element for the time-dependent protein binding (Figure 5d). The ability of silica to form a biocorona was also shown in other studies [22]. Ma et al. [37] showed a similar adsorption tendency of three proteins (human serum albumin, ϒ-globulin and fibrinogen) on mesoporous silica NPs obtained by ammonia base-catalyzed method, the process being especially rapid within the first 10 min, and then increasing slowly for the next 30 min. In comparison, a study on TiO_2_ NPs showed the lowest adsorption for HSA compared to ϒ-globulins and fibrinogen [38], the highest quantity adsorbed being observed for the smallest NPs, partly due to the larger specific surface area and increased available adsorption sites. 

The nanoscale surface topographies created by NPs agglomeration in clusters, as confirmed by SEM images, can significantly influence the kinetics of protein adsorption. Dynamic interactions between these surfaces and proteins are very complex due to the combination of attractive and repulsive forces that are triggered by local modifications in surface properties. Taking into account that the lowest amount of albumin was adsorbed on TiO_2_ NPs, which created a very complicated 3D surface as revealed on SEM images, we could affirm that less protein was adsorbed on nano-rough surfaces (as previously reviewed [39]), compared to more uniform topographies as in the case of SiO_2_ NPs. 

A good correlation was observed for results obtained by SDS-PAGE and FTIR measurements. Both analyses showed a higher adsorption of BSA on the surface of NPs compared to HSA. In addition, a time-dependent increase in HSA adsorption during the incubation was noticed for SiO_2_ NPs. 

The molecular simulations studies revealed that BSA is strongly adsorbed on silica NPs by the effects of screening ions, the surface water layers and hydrophobic forces, with 8 H-bonds formed between it and the surface water layers, BSA being able to diffuse across the silica surface [40]. The analysis of BSA adsorption on amorphous SiO_2_ NPs revealed that coverage with this protein was the greatest at the isoelectric point of the BSA-SiO_2_ complex with a value of ca. 3 ± 1 × 10^11^ molecules cm^−2^ [41]. A previous study regarding the interaction of BSA with TiO_2_ (~22 nm) and SiO_2_ (~14 nm) NPs noticed a higher coverage on TiO_2_ NPs, the BSA being completely unfold on TiO_2_ NPs and having an extended conformation on SiO_2_ NPs [42].

Furthermore, it was proved that BSA adsorption on TiO_2_ highly depends on pH as well as the presence of salts, which affect protein conformational change upon adsorption [43]. For vapor-phase-grown TiO_2_ NPs, Marquez et al. [44] demonstrated that BSA adsorbs only at the outer agglomerate surfaces without penetrating the interior of the agglomerates, and the adsorption-induced protein conformational change is associated with a decrease in the helical content. Furthermore, a decrease of % β-sheets by increasing the particle size was observed, the structural deformations being more evident for small particle sizes due to the increased interaction with highest surface area [45].

## 5. Conclusions

Our results showed that the two types of albumin adsorbed on the surface of both types of NPs, but in different quantities. A higher adsorption of BSA on the surface of NPs was observed compared to HSA, and a time-dependent increase in albumin’s adsorption during the incubation was obtained for SiO_2_ NPs. In addition, we noticed different changes in the structure of albumins depending on the physicochemical properties of each type of particles tested. Differences in the amide I/II bands ratios, correlated with similar shifts in amide I and amide II bands’ central frequencies, suggest conformational transitions when the proteins bind to these NPs. In conclusion, our study provides a comparative analysis between the different characteristics of NPs and the protein corona formed on the particle surface and effects induced on protein structure in order to direct the development of “safe-by-design” NPs, as their demands for research and applications continue to increase. However, we are not yet able to predict how the synthetic identity of NPs influences the structure, composition and evolution of the protein corona, but the knowledge is still growing in this way.

## Figures and Tables

**Table 1:** Characterization of SiO_2_ and TiO_2_ NPs by hydrodynamic size, polydispersion index (PdI) and zeta potential measurements. The values shown are averages of three measurements ± standard deviation.

Nanoparticles	Hydrodynamic Size (d.nm)	PdI	Zeta Potential (mV)
SiO_2_	134.4 ± 1.431	0.255 ± 0.009	−24.4 ± 0.929
TiO_2_	1307 ± 48.01	0.433 ± 0.086	−12.8 ± 0.643

**Table 2:** The wavenumbers associated to the central frequencies of pure NPs, pure proteins and proteins incubated with NPs for different periods of time.

Sample	Central Frequency (cm^−1^) SiO_2_ Nanoparticles			Central Frequency (cm^−1^) TiO_2_ Nanoparticles		
	Amide I	Amide II	SiO_2_ I	Amide I/ TiO_2_ I	Amide II	TiO_2_ II
SiO_2_ NP	–	–	1078	–	–	–
TiO_2_ NP	–	–	–	1637.5	–	409
BSA	1643	1530	–	1643	1530	–
BSA_NP_10’	1651	1549	1074	1651	1541	411
BSA_NP_30’	1651	1543	1076	1649	1539	409
BSA_NP_60’	1651	1541	1074	1649	1539	409
HSA	1645	1531	–	1645	1531	–
HSA_NP_10’	1649	1548	1072	1651	1541	405
HSA_NP_30’	1649	1545	1074	1651	1541	409
HSA_NP_60’	1649	1545	1072	1649	1539	409
